# Post Transplantation Cyclophosphamide Improves Outcome of Autologous Hematopoietic Stem Cell Transplantation in Animal Model of Multiple Sclerosis

**DOI:** 10.1007/s00005-021-00619-4

**Published:** 2021-06-28

**Authors:** Kaja Kasarełło, Emilian Snarski, Dorota Sulejczak, Tomasz Ciesielski, Agnieszka Wiśniewska, Robert Wrzesień, Agnieszka Cudnoch-Jędrzejewska

**Affiliations:** 1 Chair and Department of Experimental and Clinical Physiology, Laboratory of Centre for Preclinical Research, Medical University of Warsaw, Banacha 1B, 02-097 Warsaw, Poland; 2 Department of Experimental Pharmacology, Mossakowski Medical Research Centre, Warsaw, Poland; 3 Department of Laboratory Medicine, Medical University of Warsaw, Warsaw, Poland; 4 Central Laboratory of Experimental Animals, Medical University of Warsaw, Warsaw, Poland

**Keywords:** Experimental allergic encephalomyelitis, Multiple sclerosis, Hematopoietic stem cell transplantation, Post-transplantation cyclophosphamide

## Abstract

Experimental allergic encephalomyelitis (EAE) is the animal model of multiple sclerosis (MS). Autologous hematopoietic stem cell transplantation (AHSCT) has recently been recognized as the standard treatment for MS. The aim of our experiment was to investigate the effect of AHSCT with the addition of low-dose post-transplantation cyclophosphamide (Cy) on EAE in rats. Low dose post-transplantation Cy is used in haploidentical HSCT to reduce the risk of graft versus host disease. We hypothesized that it could bring additional benefit in autologous HSCT in autoimmune diseases. Rats with evoked EAE were treated with high dose (125 mg/kg) Cy, followed by AHSCT or high dose (125 mg/kg) Cy followed by AHSCT followed by low dose (20 mg/kg) Cy in two-time schedules—with the therapy applied during the pre-symptomatic or symptomatic phase of the disease. Both AHSCT and AHSCT with post-transplantation Cy in accordance with both time schedules reduce the intensity of the inflammatory response in the CNS, in comparison with non-treated EAE rats. The reduction of clinical symptoms was present in all AHSCT treatment protocols, however, it was significantly stronger when post-transplantation Cy was given during the symptomatic phase of the disease. AHSCT with the addition of post HSCT low dose Cy improved the results of AHSCT by not only reducing the intensity of inflammation in the CNS but also by significantly reducing the clinical symptoms in treated animals when compared to AHSCT alone. We provide an experimental rationale that the addition of post-transplantation Cy may improve the outcome of HSCT in MS.

**Graphic Abstract:**

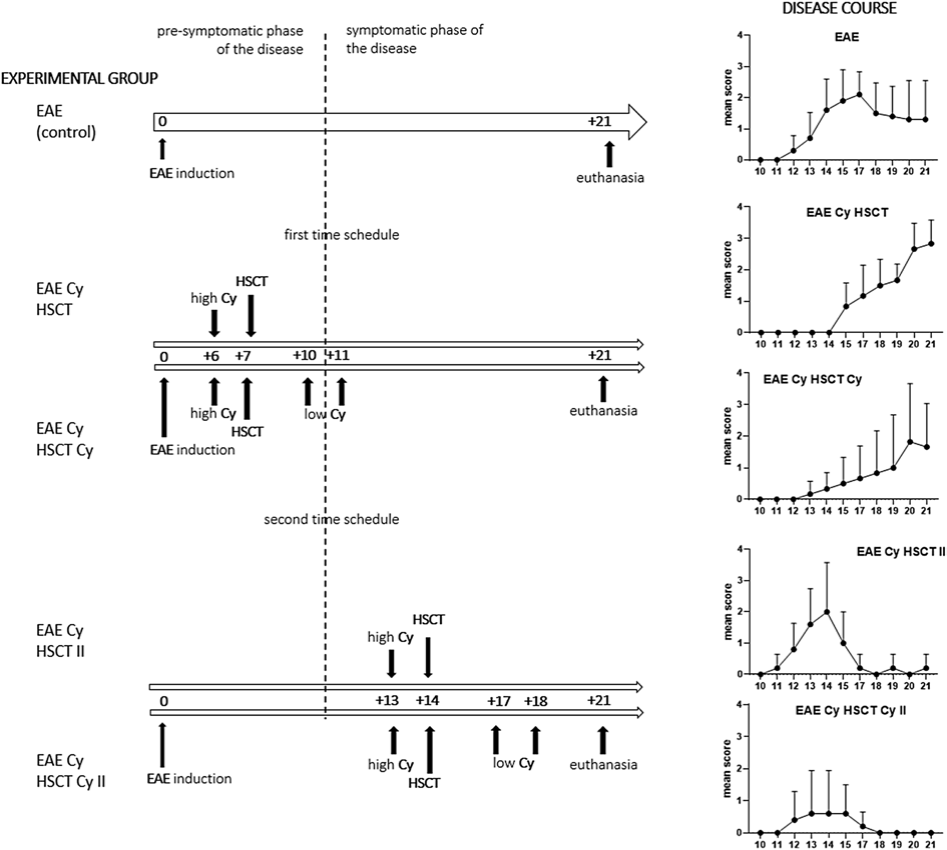

## Introduction

Multiple sclerosis (MS) is a human autoimmunological disease affecting over 2.5 million people worldwide (Hunter 2016). MS leads to progressive physical disability worsening the quality of life. In MS, autoreactive T cells are activated in the periphery (bystander suppression, molecular mimicry, etc.) and migrate through an opening of the blood–brain barrier into the CNS. The T cells recognize myelin antigens in the CNS and an inflammatory response is generated. During the inflammation, the surrounding tissue is also destroyed by an ongoing process, eventually leading to the loss of axons and whole neurons (Grigoriadis and van Pesch 2015; Sospedra and Martin 2005).

Recently, autologous hematopoietic stem cell transplantation (AHSCT) has been recognized as an option for the standard treatment in selected patients with MS and systemic sclerosis (Cohen et al. 2019; Kowal-Bielecka et al. 2017; Sharrack et al. 2020). This method is based on the use of immunoablative or myeloablative treatment and the transplantation of a patient’s own hematopoietic stem cells. After mobilization with chemotherapy and/or granulocyte-colony stimulating factor (G-CSF), hematopoietic stem cells are collected from the patient. Next, pre-transplantation conditioning, with a high dose of a chemotherapeutic agent (one or a combination of agents) or radiation is given for immuno- or myeloablation. Afterwards, the transplantation of autologous hematopoietic stem cell is performed. This procedure can lead to an improvement of the clinical condition and long-lasting remission of MS, but not all patients benefit—especially those with progressive forms of MS (Atkins et al. 2016; Burt et al. 2019). The CD34+ selection of grafts has not been tested in a randomized study in AHSCT in MS but the available data do not show an improvement of results by this graft modification (Nash et al. 2015). The rate of serious complications connected with AHSCT in autoimmune diseases is relatively low compared with other diseases—in a recent series of 1000 patients, the mortality rate was 0.2% and serious adverse events were observed in a few percent of cases (Murrieta-Álvarez et al. 2021).

Post-transplantation treatment has been unexplored in this setting. Post-transplantation cyclophosphamide (Cy) is used in haploidentical HSCT to suppress graft versus host disease (Williams et al., 2020). The use of Cy on days +3 and +4 enables the efficient elimination of activated autoreactive lymphocytes. It has not yet been tested whether this approach confers possible additional benefits in an AHSCT setting in autoimmune diseases.

We hypothesized that the addition of post-transplantation Cy in MS might improve the effect of AHSCT against autoimmunity. Moreover, additional effects on the immune system might be possible as the Cy might destroy the T cell clones remaining after AHSCT or active clones transferred with the hematopoietic stem cell graft. To investigate this strategy, we used rats with the evoked animal model of MS—experimental allergic encephalomyelitis (EAE) (Procaccini et al. 2015; Young and Welsh 2008). AHSCT has not been tested in this model—neither with Cy as a pre-conditioning agent nor as a post-conditioning agent.

## Material and Methods

The experiments were performed with 12-week old Lewis female rats (*n* = 62). The rats were obtained from the breeding facility of the Mossakowski Medical Research Centre. The experiments were performed based on the consent of the Local Ethics Committee (Act No. 494/2018 and WAW2/129/2018). The guidelines of the EU Directive 2010/63/EU were followed for the animal experiments.

### Induction of EAE

All experiments were related to the day post-immunization (DPI). At day 0 DPI, the rats were subjected to anesthesia with an intraperitoneal injection of a mixture of 100 mg/kg ketamine and 10 mg/kg xylazine (Vetoquinol, Biowet). Next, 100 µl of the immunization mixture was injected subcutaneously at the lower left and right back of the animal (100 µl at each site). The mixture consisted of Freund’s Complete Adjuvant (DIFCO Laboratories) with 50% guinea pig spinal cord homogenate in phosphate-buffered saline (PBS) (Merck Millipore), 1:1, supplemented with *Mycobacterium tuberculosis* (DIFCO Laboratories), 1 mg/ml of the mixture.

The bodyweight of the animals was measured daily or every second day and clinical symptoms were evaluated starting on 10 DPI, using a 5-grade scale where 0—no symptoms, 1—limp tail, 2—hind limb paresis, 3—incontinence, paraplegia, 4—quadriplegia, 5—death. The number of days with clinical symptoms was counted.

The mean score for the evaluated clinical symptoms was counted. The body mass ratio (mass at the day of euthanasia/mass at day 0) was calculated. The percentage of days with clinical symptoms observed from 10 DPI was counted. The mean score, the body mass ratio, and the percentage of days with clinical symptoms observed were presented graphically.

### Conditioning and Autologous Hematopoietic Stem Cell Transplantation (AHSCT)

The pre-transplantation dose of Cy was adjusted as follows: 12 Lewis female rats were divided into four groups receiving a single dose of 50, 75, 100, or 125 mg/kg of Cy (Baxter) intraperitoneally. The white blood cell (WBC) count in the full blood sample collected from the tip of the tail was analyzed using fluorescent flow cytometry (Sysmex XN2000, Sysmex, Japan) 4 and 7 days after the Cy injection. The lowest possible dose of Cy which was capable of removing all leukocytes from the blood to minimize the toxic effect of Cy was specified as 125 mg/kg (Table 1). Cy at this dose was later used as a conditioning agent prior to AHSCT.

**Table 1:** White blood cell (WBC) count in rats

Rat	Cyclophosphamide dose	Day 4	Day 7
		WBC count	WBC count
1	50 mg/kg	5000/μl	5000/μl
2		6000/μl	2000/μl
3		5000/μl	2000/μl
4	75 mg/kg	2000/μl	3000/μl
5		1000/μl	3000/μl
6		1000/μl	1000/μl
7	100 mg/kg	1000/μl	2000/μl
8		1000/μl	1000/μl
9		0/μl	0/μl
10	125 mg/kg	0/μl	2000/μl
11		0/μl	0/μl
12		0/μl	0/μl

Bone marrow was obtained from the donor rats from the same litter to maximize the genetic similarity and mimic AHSCT. To obtain hematopoietic stem cells, six donor rats were subjected to anesthesia with an intraperitoneal injection of a mixture of 100 mg/kg ketamine and 10 mg/kg xylazine. Next, the rats were sacrificed by decapitation and the long bones were isolated from the limbs. Next, the capitula were removed and the intramedullary canals were flushed with the smallest volume of PBS possible to avoid overdilution of the cell suspension. Next, the cell suspension was filtered using a cell strainer (70 µm, Corning Life Sciences) to remove debris. The cells were counted (Sysmex XN2000, Sysmex, Japan) and diluted to obtain a 20 × 10^6^ cell suspension in 0.5 ml of PBS. Each portion of cells was prepared for immediate transplantation by placing them in syringes.

Twenty-four hours before AHSCT, all animals undergoing the procedure received a single intraperitoneal injection of a high dose, i.e. 125 mg/kg Cy. The hematopoietic stem cells were transplanted by an intravenous injection of a 20 × 10^6^ cell suspension in 0.5 ml of PBS. On the third and fourth day after AHSCT, half of the rats received an additional 20 mg/kg of intraperitoneal Cy.

**Fig. 1:**
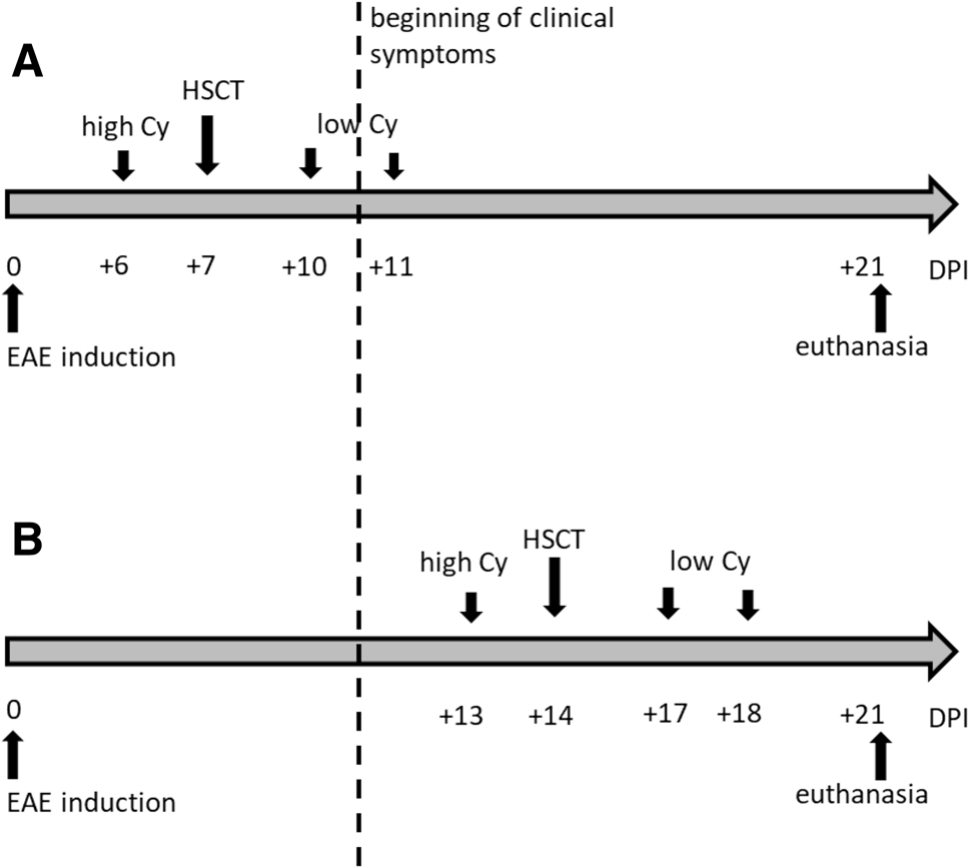
Time schedule of experiment. **A** First (I) schedule where: conditioning at 6 DPI, HSCT at 7 DPI, low dose cyclophosphamide at 10 DPI and 11 DPI. **B** Second (II) schedule where: conditioning at 13 DPI, HSCT at 14 DPI, low dose cyclophosphamide at 17 DPI and 18 DPI, high Cy: conditioning with cyclophosphamide, low Cy: low dose cyclophosphamide

There were two-time schedules in the study. In the first schedule (I), conditioning was given at 6 DPI, AHSCT was performed at 7 DPI, and low dose post-transplantation Cy was given at 10 DPI and 11 DPI. In the second schedule (II), conditioning was given at 13 DPI, AHSCT was performed at 14 DPI, and low dose post-transplantation Cy was given at 17 DPI and 18 DPI (Fig. 1.).

The control animals were healthy non-treated/treated rats with HSCT with post-transplantation Cy or with evoked EAE without treatment. The number of animals in the experimental groups (*n*) varied between 5 and 10.

### Tissue Isolation

The rats were euthanized on the 21st day after evoking EAE. After inducing anesthesia with an intraperitoneal injection of a mixture of 100 mg/kg ketamine and 10 mg/kg xylazine, blood was collected directly from the heart and then the rats were decapitated and the spinal cord (the cervical and thoracic parts) was isolated. The spinal cords were fixed for 24 h in 4% formaldehyde in PBS and saturated with sucrose by immersing in 10, 20, and 30% (w/v) sucrose solutions in PBS. Next, the spinal cords were frozen at −80 °C.

### Histopathological Analysis

The frozen spinal cords were embedded with tissue freezing medium (Leica, Germany) and immediately cut on 20 μm-thick glass-mounted sections using a cryostat (Leica, Germany). Salinized glass microscope slides (VWR) were used. Finally, the sections were dried at room temperature, stained with hematoxylin and eosin in accordance with the routine procedure and covered with DPX Mountant for histology (Sigma-Aldrich, Germany). Next, the sections were examined and captured with a light microscope (Nikon, Japan) equipped with a CCD camera (Nikon) and an image analysis system making it possible to photograph microscopic material. The scientist who evaluated the labelled sections were blinded to the identity of the samples. The percentage area occupied by inflammatory cell infiltration on each individual spinal cord section was measured using ImageJ (Fiji edition) software.

### Statistical Analysis

The distribution of continuous variables was evaluated with the Shapiro–Wilk test; for normally distributed variables the mean and the standard deviation (SD) were reported, otherwise the median and the 25th and 75th percentile (Q1 and Q3) were calculated. The normally distributed continuous variables were compared with the ANOVA test, otherwise the Kruskal–Wallis test was used. The significance level was set at 0.05. The statistical significance was marked on the figures as follows: *p < 0.05; **p < 0.01; ***p < 0.001; ****p < 0.0001.

## Results

### Body Mass

**Fig. 2:**
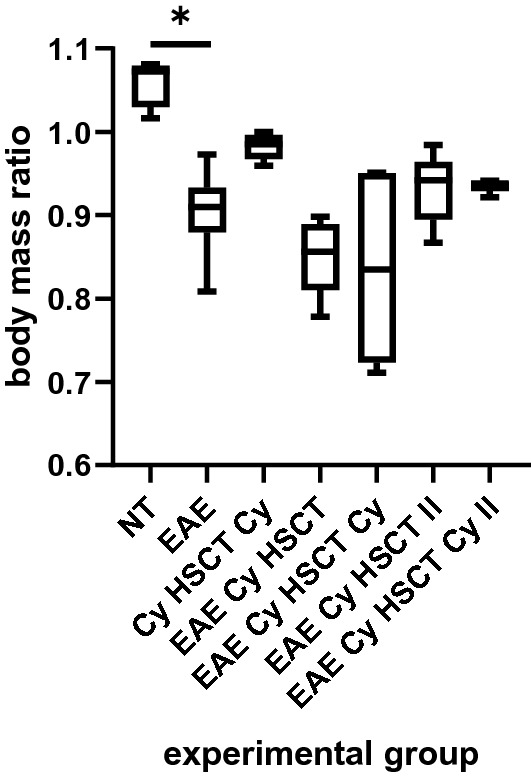
Body mass ratio. EAE: experimental allergic encephalomyelitis, Cy: cyclophosphamide, HSCT: hematopoietic stem cell transplantation, NT: non-treated animals, II: for the second schedule of treatment

Changes in the body mass ratio were observed in all groups of animals (Fig. 2). The non-treated animals (NT) increased their body mass during the experiment, whereas the procedures performed on the animals had a tendency to decrease their body mass. A significant drop in body mass was observed in non-treated animals with EAE evoked (EAE) in comparison with non-treated animals (NT). In the healthy control animals treated with AHSCT with post-transplantation Cy (Cy HSCT Cy), a slight decrease in the body mass ratio was observed. In EAE animals treated with the procedure in accordance with the first time schedule, both without (EAE Cy HSCT) and with post-transplantation Cy (EAE Cy HSCT Cy), the tendency to decrease the body mass ratio was strongest. In EAE animals treated in accordance with the second time schedule, both without (EAE Cy HSCT II) and with post-transplantation Cy (EAE Cy HSCT Cy II), a lower tendency to decrease the body mass was observed than in animals treated in accordance with the first time schedule. There were no differences between EAE animals treated with AHSCT and animals treated with AHSCT and additional low dose Cy neither in accordance with the first time schedule (EAE Cy HSCT vs. EAE Cy HSCT Cy) nor in accordance with the second time schedule (EAE Cy HSCT II vs. EAE Cy HSCT Cy II).

### Clinical Symptoms

**Fig. 3:**
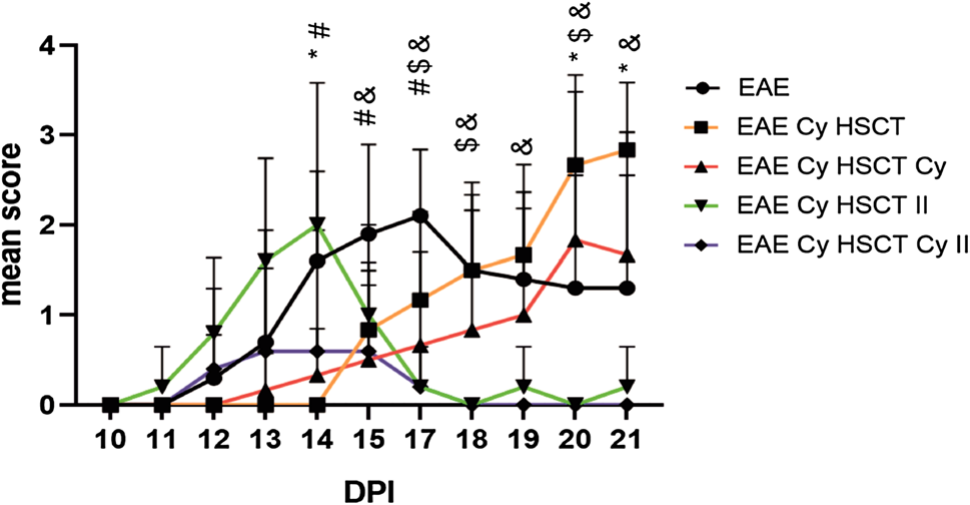
Mean score during the disease progression observed in animals. EAE: experimental allergic encephalomyelitis, Cy: cyclophosphamide, HSCT: hematopoietic stem cell transplantation, II: for the second schedule of treatment. Statistical significance marks: * for EAE versus EAE Cy HSCT; # for EAE versus EAE Cy HSCT Cy; $ for EAE versus EAE Cy HSCT II; & for EAE versus EAE Cy HSCT Cy II

Figure 3 presents the mean score of disease symptoms observed in animals during the disease course, beginning with 10 DPI up to the day of euthanasia of the animals at 21 DPI. The disease course in non-treated animals with EAE evoked (EAE) presents the onset of symptoms at 12 DPI with the peak of symptoms at 17 DPI followed by a progressive regression of symptoms. For EAE animals treated with AHSCT (EAE Cy HSCT) and AHSCT followed by a low dose of Cy (EAE Cy HSCT Cy) in accordance with the first time schedule, a delay of the onset of symptoms was observed up to 13 DPI in the EAE Cy HSCT Cy group and up to 15 DPI in the EAE Cy HSCT group. The mean score was significantly lower in the treated animals than in the EAE non-treated animals (EAE) at 14 DPI in animals treated with AHSCT (EAE Cy HSCT) and at 14 DPI to 17 DPI in animals treated with AHSCT followed by a low dose of Cy (EAE Cy HSCT Cy). Afterwards, slow disease progression with increasing symptoms was observed with a peak of symptoms at 20 DPI and 21 DPI in the EAE Cy HSCT Cy and EAE Cy HSCT groups, respectively, and the mean score was higher than in EAE non-treated animals. The mean score at the peak of symptoms (20 DPI and 21 DPI) in the group of animals treated with AHSCT (EAE Cy HSCT) was significantly higher than in EAE non-treated animals (EAE). Additionally, the mean score values observed at the peak of symptoms in animals treated with AHSCT (EAE Cy HSCT) was even higher than in EAE non-treated animals (EAE).

In EAE animals treated in accordance with the second time schedule, the onset of clinical symptoms was similar to the onset of clinical symptoms in non-treated EAE animals (EAE) and was observed at 11 DPI for animals treated with AHSCT (EAE Cy HSCT II) and at 12 DPI in animals treated with AHSCT followed by low dose Cy (EAE Cy HSCT Cy II). The peak of symptoms was observed at 14 DPI in animals treated with AHSCT (EAE Cy HSCT II) and from 13 to 15 DPI in animals treated with AHSCT followed by low dose Cy (EAE Cy HSCT Cy II). Afterwards, the mean score observed in both groups of EAE animals treated in accordance with the second time schedule (EAE Cy HSCT II and EAE Cy HSCT Cy II) dropped and was significantly lower than in non-treated EAE animals (EAE) at 17 DPI, 18 DPI, and 20 DPI for animals treated with AHSCT (EAE Cy HSCT II) and from 15 DPI up to the end of the experiment for animals treated with AHSCT followed by low dose Cy (EAE Cy HSCT Cy II), who showed no symptoms from 18 DPI.

The clinical disease course was very similar for EAE animals treated with AHSCT both without and with post-transplantation Cy in accordance with the first time schedule (EAE Cy HSCT and EAE Cy HSCT Cy) and also with the second time schedule (EAE Cy HSCT II and EAE Cy HSCT Cy II).

**Fig. 4:**
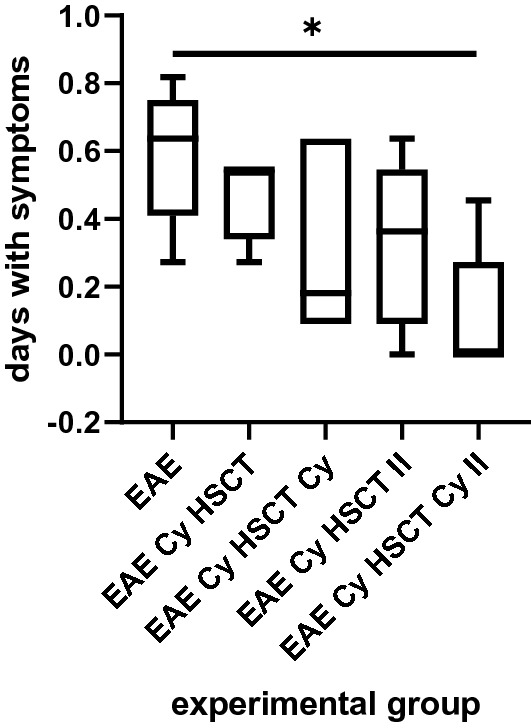
Number of days with clinical symptoms present in the experimental animals. EAE: experimental allergic encephalomyelitis, Cy: cyclophosphamide, HSCT: hematopoietic stem cell transplantation, II: for the second schedule of treatment

Figure 4 presents the number of days with clinical symptoms observed in the experimental animals during the disease course beginning at 10 DPI up to the end of the experiment at 21 DPI. In EAE animals treated with protocols in accordance with both time schedules and both with or without the additional low dose Cy (EAE Cy HSCT, EAE Cy HSCT Cy, EAE Cy HSCT II, EAE Cy HSCT Cy II), a decreasing tendency in the number of days with symptoms was observed in comparison with non-treated EAE animals (EAE). It reached statistical significance in the group of animals treated with AHSCT followed by low dose Cy in accordance with the second time schedule protocol (EAE Cy HSCT Cy II) in comparison with non-treated EAE animals (EAE). A decreasing tendency was observed in the number of days with symptoms in animals treated with the additional low dose of Cy both in accordance with the first (EAE Cy HSCT Cy) and second (EAE Cy HSCT Cy II) time schedule protocols in comparison with the animals treated with AHSCT (EAE Cy HSCT and EAE Cy HSCT II).

### Histopathological Analysis

**Fig. 5:**
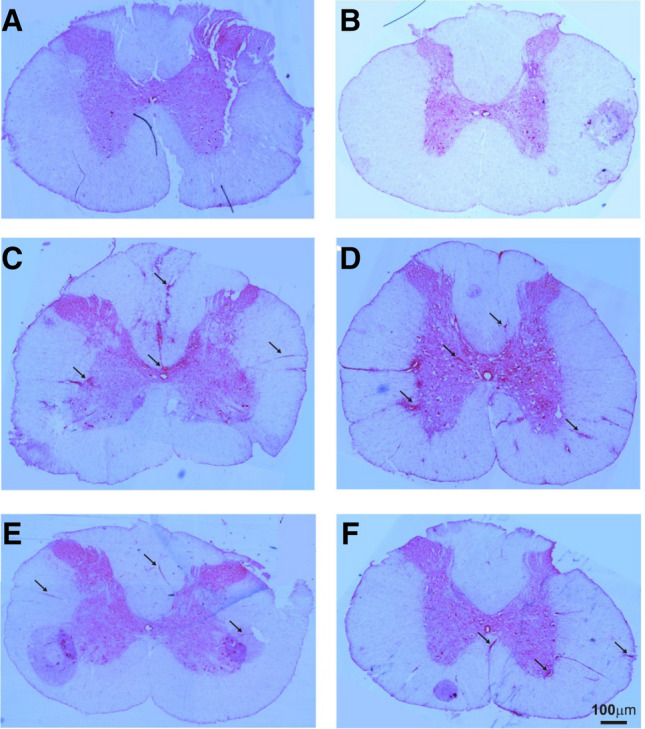
Morphological changes. Spinal cord cross-section. Hematoxylin and eosin staining. **A**, **B** Sections of the cervical and thoracic spinal cord of the control animals, respectively. **C**, **D** Sections of the cervical and thoracic spinal cord of EAE non-treated animals, respectively. **E**, **F** Sections of the cervical and thoracic spinal cord of EAE treated animals, respectively. Inflammatory infiltrations marked by arrows

Hematoxylin and eosin staining made it possible to observe the spinal cord structure, with visible white and grey matter forming the dorsal and ventral horns. Sections obtained from non-treated healthy animals (NT), from healthy animals treated with AHSCT, and post-transplantation Cy (Cy HSCT Cy) control animals showed the proper morphology and no inflammatory infiltrations in the cervical (Fig. 5A) and thoracic (Fig. 5B) spinal segments. In the sections obtained from non-treated EAE rats (EAE), numerous inflammatory infiltrations both in the grey and white matter were present in the cervical (Fig. 5C) and thoracic (Fig. 5D) spinal cord segments. Sections obtained from EAE rats, treated with protocols in accordance with both time schedules, and without the use of additional low dose Cy (EAE Cy HSCT, EAE Cy HSCT Cy, EAE Cy HSCT II, EAE Cy HSCT Cy II) were very similar, showing a few areas of inflammatory infiltrations mostly located in the white matter of both cervical (Fig. 5E) and thoracic (Fig. 5F) spinal cord segments.

**Fig. 6:**
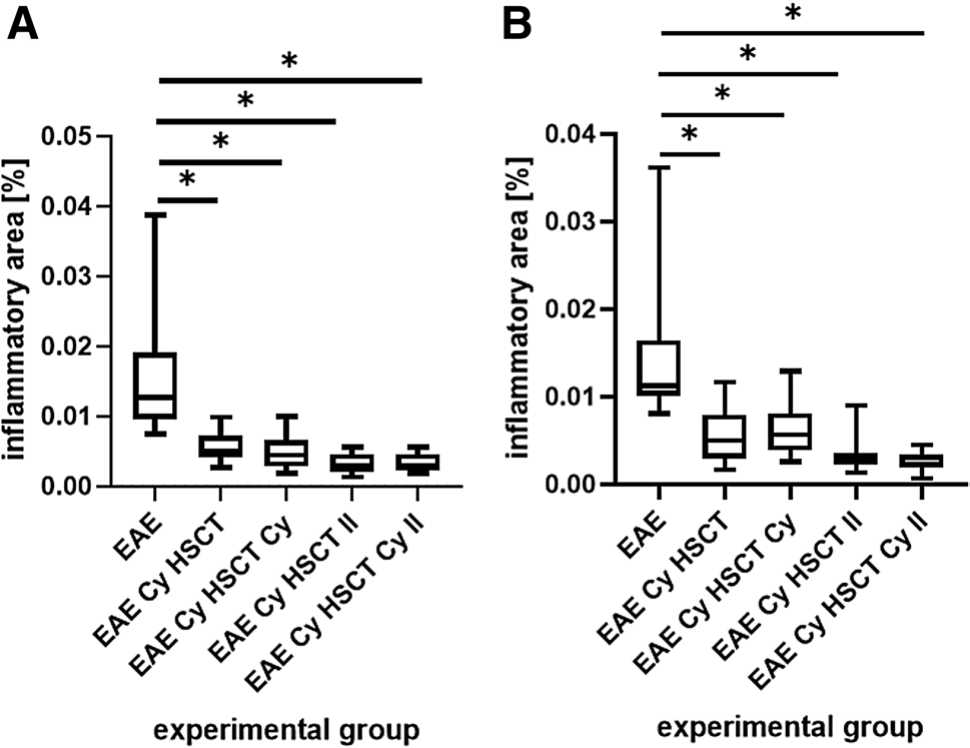
Percentage of the area of cervical (**A**) and thoracic (**B**) spinal cord section occupied by inflammatory cell infiltration. EAE: experimental allergic encephalomyelitis, Cy: cyclophosphamide, HSCT: hematopoietic stem cell transplantation, II: for the second schedule of treatment

Morphometric analysis was performed in both cervical and thoracic spinal cord segments. Figure 6A presents the percentage of the area of the cervical spinal cord infiltrated by inflammatory cells. The results obtained showed that treatment of the EAE animals with protocols in accordance with both time schedules and with or without the use of additional low dose Cy (EAE Cy HSCT, EAE Cy HSCT Cy, EAE Cy HSCT II, EAE Cy HSCT Cy II) significantly reduced the number and size of areas occupied by inflammatory infiltrations in comparison with non-treated EAE animals (EAE). Similarly, the area infiltrated by inflammatory cells within the thoracic spinal cord (Fig. 6B) was significantly decreased in EAE treated animals (EAE Cy HSCT, EAE Cy HSCT Cy, EAE Cy HSCT II, EAE Cy HSCT Cy II) in comparison with EAE non-treated animals (EAE). There were no differences observed in the area of the section occupied by inflammatory infiltrations between EAE animals treated with AHSCT and animals treated with AHSCT and additional low dose Cy in accordance with the first time schedule (EAE Cy HSCT vs. EAE Cy HSCT Cy) or with the second time schedule (EAE Cy HSCT II vs. EAE Cy HSCT Cy II).

## Discussion

The efficiency of AHSCT in MS has been demonstrated in randomized clinical trials (Burt et al. 2019; Mancardi et al. 2015). However, there is to date no preclinical data on how to improve the results of AHSCT with modifications to the chemotherapy given before and after. With this aim, we established an animal model of AHSCT in MS. With that model, we showed that a very simple modification of the procedure by the addition of Cy post AHSCT is well tolerated and possibly adds the benefit of a reduction of clinical symptoms. To our knowledge, this is the first study that shows that such a modification could improve the results of AHSCT in an autoimmune disease.

This study provides the experimental background that could be used for establishing a similar procedure in patients—moving a part of the Cy from conditioning into post HSCT treatment. The study of the splitting of the Cy dose into premobilization and conditioning shows very promising results (Ruiz-Argüelles et al. 2019, 2017). The approach presented in our study might help to maintain the dose density of chemotherapy and possibly improve the clinical outcome.

The design of the experiment assumed the use of two-time schedules. In the first time schedule, the immunoablative dose of Cy followed by HSCT was given during the pre-symptomatic phase of the disease. In the second time schedule, the above procedure was performed after the onset of the symptoms. It was clearly shown that the disease symptoms were delayed with the use of the first time schedule and that the disease symptoms were dramatically decreased with the use of the second time schedule. Our results are similar to those obtained by Karussis et al. (1992). They induced chronic-relapsing EAE in SJL/J mice, who were treated with 300 mg/kg Cy followed by syngeneic bone marrow transplantation (depleted from T cells) in the pre- or symptomatic phase of the disease and observed a delay or a great decrease of symptoms, respectively. Additionally, after the second immunization, a lower number of treated animals relapsed and the clinical symptoms decreased. In our experiment, when the HSCT was performed in the pre-symptomatic EAE phase the mean clinical score obtained at the peak of symptoms was even higher than in EAE non-treated rats. However, the dose of pre-transplantation Cy used in the experiments of Karussis et al. was 300 mg/kg, which is very high with strong potential cytotoxicity. It was shown that a dose of 300 mg/kg Cy without following bone marrow transplantation was lethal to 30% of the animals (Karussis et al. 1992). In another experiment, when 350 mg/kg Cy was used, all of the mice died, but when the dose was followed by bone marrow transplantation, the effect was totally reversed (Karussis et al. 1999). In another experiment, when conditioning of 200 mg/kg Cy with total body irradiation (TBI) at a dose of 500 cGy was performed in C57BL/6J mice prior to syngeneic bone marrow transplantation at the peak of clinical symptoms, there was a significant decrease of clinical score in comparison with EAE non-treated mice, but the result was delayed in time and the symptoms were still present (Meng et al. 2011).

In our study, a similar disease course was observed in both time schedules, irrespective of whether the additional post-transplantation low dose Cy was given or not. However, both in the first and in the second time schedule, a tendency was observed for clinical symptoms to improve when the additional post-transplantation Cy was given. The clinical symptoms were significantly reduced when HSCT was performed during the symptomatic phase of the disease. The result was stronger when the additional post-transplantation Cy was given—not only was the peak of symptoms minimal but also a significant decrease of symptoms was observed afterwards on every day of observation.

We also compared the total number of days with clinical symptoms observed in rats. In both time schedules, if additional post-transplantation Cy was given to the rats then there was a tendency for the number of days with symptoms to decrease. But only procedures performed in the symptomatic phase of the disease with additional low dose Cy ensured a significant drop in the number of days observed with symptoms. These results clearly showed that the symptoms were minimized almost to zero when AHSCT was performed during the symptomatic phase of EAE, which can be considered as mimicking MS relapse in patients.

EAE and the treatment protocols impacted the body mass of the animals. The control non-treated animals gained weight during the experiment, while all of the procedures resulted in a decrease in body mass (EAE and all AHSCT procedures). Evoking EAE caused the largest body mass reduction, which was significant, while applying therapy had a tendency to decrease the body mass reduction. Animals treated with AHSCT and post-transplantation Cy, which was potentially the most toxic, demonstrated the lowest body mass reduction after treatment once the autoimmune reaction was established.

Histopathological analysis, aiming to show the intensity of inflammatory infiltrations in the cervical and thoracic spinal cord, revealed that all combinations of therapies used in the study significantly decreased the intensity of the immune reaction. These observations are consistent with those obtained by Karussis et al. showing a decrease in infiltrations in the brain sections in mice after treatment with syngeneic bone marrow transplant (Karussis et al. 1992). However, it is unclear at this stage whether, beside the benefits of post-transplantation Cy at the clinical level, there are accompanying changes in the immune cell population.

Some questions arise from our study—could post-transplantation Cy being a part of the conditioning replicate the effect of more intensive and lymphocyte-directed therapies? Could the effect of CD34+ selection be replicated with post-transplantation Cy? CD34+ selection, on the one hand, theoretically improves the results of AHSCT and, on the other hand, increases the risk of secondary autoimmunity (Atkins et al. 2016; Daikeler et al. 2011; Dubinsky et al. 2010; Handgretinger et al. 2001; Keever-Taylor et al. 2017; Oliveira et al. 2016; Ringhoffer et al. 2004). Would the allogenic HSCT also be effective in this model? This is particularly interesting as recent studies have shown that allogenic cord blood transplantation can fully abolish autoimmunity despite the lack of long-term engraftment (Burt et al. 2020). Could post-HSCT Cy also improve the results of such transplantations by reducing the risk of graft versus host disease (GvHD)? These questions might be answered by undertaking studies with the proposed HSCT model in EAE rats.

Another important issue is the possible impact of post-transplantation Cy on complications of the AHSCT procedure. The reported rates of mortality and complications in AHSCT are low (Burt et al. 2019; Mancardi et al. 2015; Murrieta-Álvarez et al. 2021; Nash et al. 2015)—increasing the toxicity by additional chemotherapy might negatively impact safety. Post Cy in MS prolongs engraftment time in haploidentical transplants so a similar effect can be expected in AHSCT (Bacigalupo and Giammarco 2019; Im et al. 2013). One approach to reduce the risk is to modify the protocol and split the doses of cyclophosphamide instead of increasing it. This approach has been tested in AHSCT in MS where the cyclophosphamide was split into a mobilization and a conditioning dose—reducing the toxicity of the approach while maintaining the effectiveness (Murrieta-Álvarez et al. 2021).

There are limitations to our study. First, despite our aim to mimic autologous transplantation by using inbred animals from the same litter as the donors, there was a small chance that there were differences between the grafts on a genetic level and this might impact the results. Second, we performed an experiment to establish a model of HSCT in EAE in the rat and focused mainly on the clinical outcomes of the disease. It will be interesting to research this field further to see whether clinical differences translate into an impact on the immune cell population regulating autoimmunological reactions.

To summarize, we established a new model for AHSCT with post-transplantation Cy in an animal model of MS that could be used for further testing of treatment strategies. With this model, we have shown that post-transplantation Cy could improve the results of AHSCT in MS.

## Data Availability

Not applicable.
